# Evaluation of the Efficiency of Active and Passive Surveillance in the Detection of African Swine Fever in Wild Boar

**DOI:** 10.3390/vetsci7010005

**Published:** 2019-12-30

**Authors:** Vincenzo Gervasi, Andrea Marcon, Silvia Bellini, Vittorio Guberti

**Affiliations:** 1 Wildlife Department, Istituto Superiore per la Protezione e la Ricerca Ambientale, 40064 Ozzano Emilia (BO), Italy; vincent.gervasi@gmail.com (V.G.); amarcon.work@gmail.com (A.M.); 2 Istituto Zooprofilattico della Lombardia ed Emilia-Romagna, 25124 Brescia, Italy; silvia.bellini@izsler.it

**Keywords:** African swine fever, wild boar, surveillance, early detection, endemic, SIR model

## Abstract

African swine fever (ASF) is one of the most severe diseases of pigs and has a drastic impact on pig industry. Wild boar populations play the role of ASF genotype II virus epidemiological reservoir. Disease surveillance in wild boar is carried out either by testing all the wild boar found sick or dead for virus detection (passive surveillance) or by testing for virus (and antibodies) all hunted wild boar (active surveillance). When virus prevalence and wild boar density are low as it happens close to eradication, the question on which kind of surveillance is more efficient in detecting the virus is still open. We built a simulation model to mimic the evolution of the host-parasite interaction in the European wild boar and to assess the efficiency of different surveillance strategies. We constructed a deterministic SIR model, which estimated the probability to detect the virus during the 8 years following its introduction, using both passive and active surveillance. Overall, passive surveillance provided a much larger number of ASF detections than active surveillance during the first year. During subsequent years, both active and passive surveillance exhibited a decrease in their probability to detect ASF. Such decrease, though, was more pronounced for passive surveillance. Under the assumption of 50% of carcasses detection, active surveillance became the best detection method when the endemic disease prevalence was lower than 1.5%, when hunting rate was >60% and when population density was lower than 0.1 individuals/km^2^. In such a situation, though, the absolute probability to detect the disease was very low with both methods, and finding almost every carcass is the only way to ensure virus detection. The sensitivity analysis shows that carcass search effort is the sole parameter that increases proportionally the chance of ASF virus detection. Therefore, an effort should be made to promote active search of dead wild boar also in endemic areas, since reporting wild boar carcasses is crucial to understand the epidemiological situation in any of the different phases of ASF infection at any wild boar density.

## 1. Introduction

African swine fever (ASF) is one of the most severe diseases of pigs and has a drastic impact on pig industry [1]. The disease is caused by the ASF virus (ASFV), which belongs to the family *Asfarviridae* and affects both domestic pigs and wild boar with a high case fatality rate. No effective vaccine or treatment exists to aid in the control of the disease. The disease is present in Africa, Europe and Asia.

In Europe, there are currently two main clusters of ASFV infection. One of them is in Sardinia where the disease was introduced in 1978 and it is caused by strains of ASFV belonging to genotype I. The second cluster is occurring in a large part of North Eastern Europe and it is caused by strains of ASFV belonging to genotype II. The latter is a highly virulent strain inducing an acute form of ASF that results in a mortality rate of 94.5–100% in both wild boar and domestic pigs [2]. In the European Union (EU), ASF was detected for the first time in Lithuania in January 2014 and since then, the disease has spread to Estonia, Latvia, Poland, Czech Republic, Hungary, Romania, Bulgaria, Belgium, Slovakia. In most of the affected areas, wild boar populations play the role of ASF virus epidemiological reservoir, maintaining indefinitely the virus in the environment, independently from any other susceptible species or vector [3]. In virgin wild boar populations, ASF is introduced either by human related activities, such as transfer of infected food or illegal trade (e.g., Czech Republic and Belgium) or through a geographical continuity of the infected wild boar or domestic pig populations (e.g., Hungary, Slovakia) [4], although in the specific cases it was not possible to trace back the specific cause of virus introduction. In wild boar, following its introduction, the virus shows an epidemic wave that tends to spread toward free areas while it remains endemic in the previously affected ones, despite the low wild boar local density resulting from virus lethality and/or control measures (hunting/culling) [4]; this pattern was observed in most of the European countries affected so far, such as Poland, Lithuania, Estonia and Latvia [4]; the virus persists in the environment since it remains viable in wild boar carcasses. The epidemiological pattern is further complicated by the presence of infected domestic pigs and the long distance transport of the virus [4]. The transportation of infected pigs and pork meat or other contaminated material is considered as the most important factor contributing to the spread of the ASF virus over long distances [4].

In the EU, ASF surveillance in wild boar addresses both early detection in free areas and the follow up of the implemented control measures in endemic areas. Passive surveillance is carried out by testing all the wild boar found sick or dead for virus detection. Active surveillance is performed by testing all hunted wild boar for virus (and antibodies) [5]. Information collected by the European Food Safety Authority (EFSA) from the Baltic countries and Poland indicates that passive surveillance provides the higher probability of early detecting ASF, although the probability has not been quantified. Indeed, most of the primary cases in wild boar were found by passive surveillance [4]. Previous studies and the examples provided by the spread of the ASF virus in the Eastern European countries have shown that the disease can persist with a very low prevalence, even when wild boar density is kept low through intensive hunting [4]. However, since the infection became endemic in many infected wild boar populations, the question on which kind of surveillance (passive or active) is more efficient in detecting the virus at low prevalence and low wild boar density is still open, in particular considering that most of the countries are eradicating ASF in wild boar through a progressive population management aimed at reducing at a very low density the infected populations; in these epidemiological situations there is a window of uncertainty where ASF virus, if still present, is hardly detected, thus making any further policy management more complicated, including a possible exit strategy addressed at re-gaining the ASF free status.

The efficiency of ASF surveillance in wild boar is determined by a combination of epidemiological parameters, by field management practices, and by the time since virus introduction. Prevalence, lethality, and recovery rates are the epidemiological parameters determining the number of affected animals to be targeted by surveillance, whereas hunting effort and carcass search patterns are the field activities which allow the collection of samples for the diagnosis of ASF in wild boar.

The aim of this study was to compare the potential efficiency of active and passive surveillance in detecting ASF genotype II in wild boar populations and in particular:

- first virus detection in a naive wild boar population (early detection);
- monitoring the epidemic period following the virus invasion of the previously ASF free wild boar population;
- monitoring the trend of the infection during the years following the introduction of the virus;

For such purpose, we built a simulation model, developed to broadly mimic the epidemiological evolution of the host-parasite interaction in the European wild boar infected populations. We analysed the results of such model to highlight the field conditions in which each detection method is more likely to provide a significant probability of early detecting the disease and monitoring it through time.

## 2. Materials and Methods

**Figure 1:**
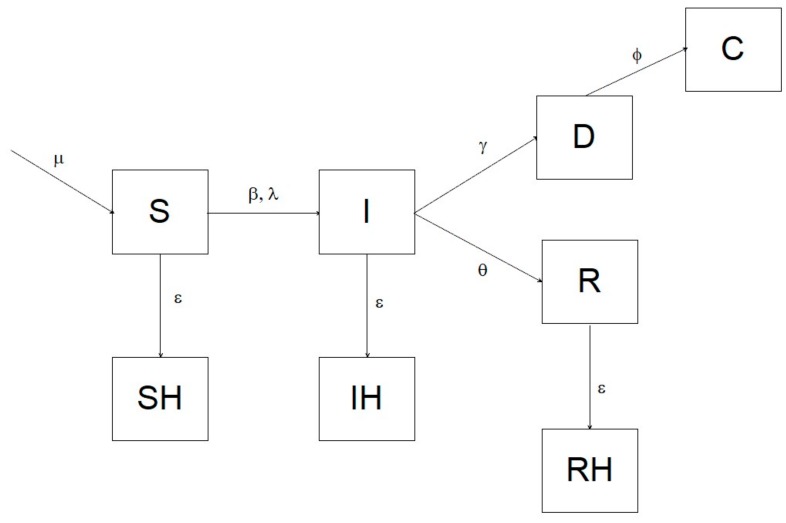
Conceptual diagram illustrating the structure of the compartmental model of the African Swine Fever (ASF) dynamics in a simulated wild boar population. The model comprised eight compartments: S = susceptible; I = infected; R = recovered; SH = susceptible hunted; IH = infected hunted; RH = recovered hunted; D = dead due to ASF; C = carcass recovered. The parameters controlling the transitions were: μ = recruitment; β = transmission rate; λ = force of the infection; ε = hunting rate; θ = recovery rate; γ = lethality; ϕ = carcass detection rate.

To assess the efficiency of different surveillance strategies in ASF endemic areas, we constructed a deterministic SIR model with a one-day step, which estimated the probability to detect the virus during the 8 years following its introduction, using both passive and active surveillance. The simulation assumed a homogeneous mixing. The model comprised eight compartments: S = susceptible; I = infected; R = recovered (immune); SH = susceptible hunted; IH = infected hunted; RH = recovered hunted; D = dead due to ASF, C = dead and recovered as carcass. We parameterized the system using an infection-induced mortality model, following Keeling and Rohani [6]. The transition of individuals from one compartment to the other is illustrated in the diagram in Figure 1, and analytically defined by the following system of differential equations:

$$\left\{\begin{array}{c} \begin{array}{c} \frac{dS}{dt}=\mu \left(S+R\right)-\beta SI-\varepsilon S\\ \frac{dI}{dt}=\beta SI-\frac{\left(\varepsilon +\theta \right)}{\left(1-\rho \right)}I \end{array}\\ \frac{dR}{dt}=\theta I-\varepsilon R\\ \frac{dSH}{dt}=\varepsilon S\\ \frac{dIH}{dt}=\varepsilon I\\ \frac{dRH}{dt}=\varepsilon R\\ \frac{dD}{dt}=\gamma I-\phi D\\ \frac{dC}{dt}=\phi D \end{array}\right.$$

In which μ is the parameter controlling the recruitment of new individuals into the susceptible compartment, β is the disease transmission rate, ε represents hunting rate, θ is the daily recovery probability (i.e., the daily probability to survive the disease and become immune), ϕ is the carcass recovery rate, ρ is the overall probability to die from ASF, and γ is the disease lethality.

**Figure 2:**
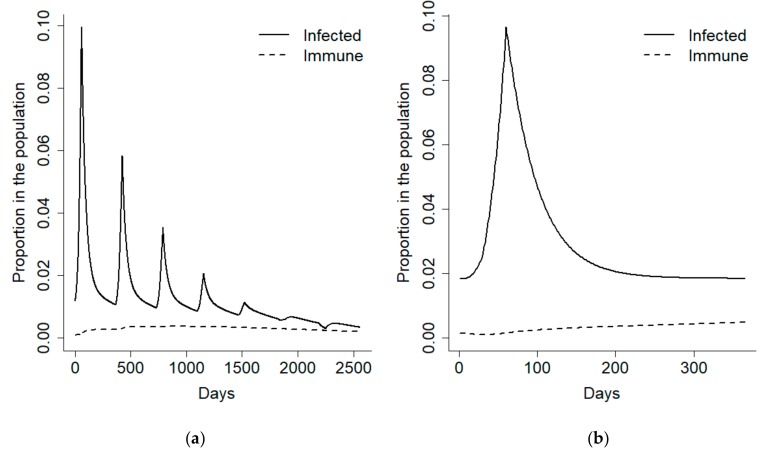
Proportion of infected and immune individuals in a simulated wild boar population affected by an African Swine Fever (ASF) epidemic during an 8-year period, as resulting from a compartmental model of the disease. The plot is illustrated for the whole 8-year period (**a**) and for just the first year (**b**).

We initially focused on the first year after the disease outbreak and divided it into an early detection phase corresponding to the 100 days following recruitment, and an epidemic phase, comprising the remaining 265 days of the year. In order to have a better control on the disease prevalence during the epidemic phase, the model was forced to obtain a 10% disease prevalence at its onset and a 2% endemic prevalence (Figure 2).

We set the yearly hunting rate to 40% of the post-reproductive population and used two different values (10% and 50%) for the proportion of carcasses recovered during the passive surveillance of ASF. We simulated three scenarios, corresponding to three wild boar populations of different size, namely 100, 400 and 1000 individuals, all living at an initial density of 1 wild boar/km^2^. The complete list of parameter values for the model is provided in Table 1.

The processes of hunting and carcass recovery were simulated accounting for their stochastic nature. For each day, we generated the number of hunted ASF positive individuals (compartment IH) from a Poisson distribution with mean equal to the number of infected individuals in that day, and probability corresponding to the daily hunting rate ε. We followed the same procedure to generate the number of immune hunted individuals which survived the infection and developed antibodies (compartment RH) and the number of carcasses recovered in any given day of the study period (compartment C). For this last randomization, we defined as available carcasses only those belonging to the individuals dead due to ASF during the last 30 days. We ran the stochastic process over 1000 iterations.

After running the model, which provided us with the absolute number of individuals in each compartment during each day of the simulated study period, we reported the total number of ASF infected carcasses recovered, and the number of antigen and antibodies positive wild boars shot during hunting. We did so for each simulated population size, and separately for the early detection (days 1–100) and epidemic periods (days 101–365). Additionally, we estimated the proportion of iterations in which the disease was detected by each of the two surveillance methods, the average day of first detection of the disease and the average time interval between two successive ASF detections.

After exploring the main model dynamics during the first year of the epidemic, we focused on assessing the relative performance of active and passive surveillance during subsequent years, and accounting for a wider range of epidemiologic and management scenarios. We re-ran the model over 1000 iterations and for a period of 8 years. This time, though, we randomly extracted the values for the main model parameters from a set of uniform distributions. We simulated the disease prevalence during the endemic phase in a range between 1 and 4%, hunting rate between 20 and 70%, carcass recovery rate between 10 and 90%, and the initial population size between 100 and 1000 individuals. After running all model iterations, we estimated the average number of runs in which the disease was detected by either active or passive surveillance each year. In addition, we pooled all model iterations in which active surveillance exhibited a higher probability to detect the disease than passive surveillance. By plotting the range of parameter values of this set of iterations, we obtained an overview of the epidemiologic and management conditions in which active surveillance was more likely to detect ASF than passive surveillance.

Finally, in order to highlight how parameter values influenced the final model output, we performed a sensitivity analysis according to Keeling and Gilligan [7]. We increased by 10% each parameter value in the model and compared the equivalent changes in the day of first ASF detection, with respect to the baseline values for both passive and active surveillance. Sensitivity values >0 and <1 indicated a less than proportional increase in the likelihood of disease detection; a sensitivity value equal to 1 indicated a true proportional increase in the likelihood of detection; sensitivity values <0 indicated that any increase in the parameter value produced a decrease in the disease detection probability.

## 3. Results

**Figure 3:**
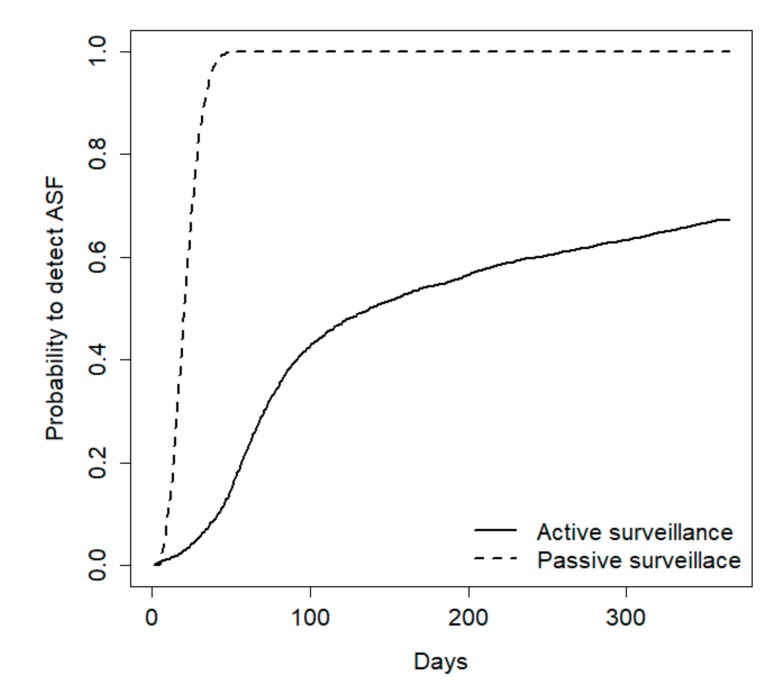
Probability to detect an African Swine Fever (ASF) outbreak with passive and active surveillance in a simulated wild boar population of 100 individuals, as resulting from a compartmental model of the disease. The plot refers to the first year after the initial disease outbreak.

During the first year, including the early detection and epidemic periods, passive surveillance was always the most effective method to detect the disease, but the relative efficacy of the two methods was strongly influenced by population size. For a population of 100 wild boars, passive surveillance exhibited a 100% probability to early detect the disease whereas hunting revealed the disease only in 43% of cases (Figure 3).

Such difference between the two strategies became less pronounced when population size increased (Table 2). Depending on population size, the day of first ASF detection when using carcass finding as detection method ranged between 1–12 days, about half of the time necessary to detect the disease when using hunting. The average number of days between two successive ASF detections was several times shorter for passive than for active surveillance (Table 2).

Overall, passive surveillance provided a much higher number of ASF detections than active surveillance during the first year following the virus invasion (Table 3). On average, the number of ASF positive carcasses found was about 10 times higher than the number of antigen or antibodies positive wild boars shot. Such difference increased to 20–40 folds when simulating a 50% carcass recovery rate (Table 3), mimicking the so-called active search of carcasses. Compared to the shooting of an infected wild boar, antibodies detection always had a negligible probability to detect the infection (Table 3).

**Figure 4:**
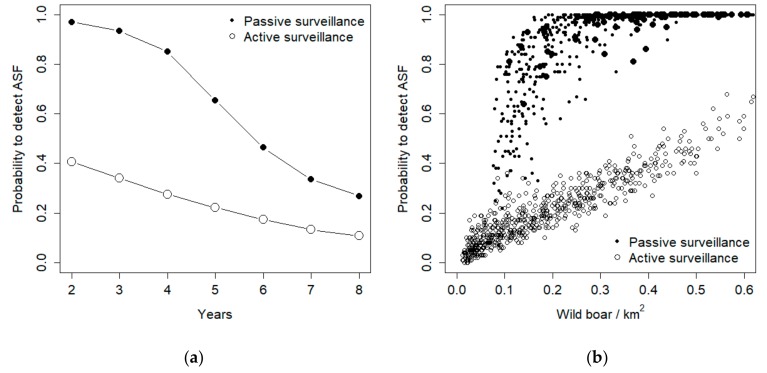
Probability to detect African Swine Fever (ASF) in a simulated wild boar population with passive and active surveillance, as resulting from a compartmental model of the disease. The plot provides the temporal trend in virus detection probability (**a**) and its relationship with population density (**b**).

During subsequent years, both active and passive surveillance exhibited a decrease in their probability to detect ASF (Figure 4a). Such decrease, though, was more pronounced for passive surveillance, so that in the last year the two methods exhibited similar performances, corresponding to about 20% success rate in detecting the disease (Figure 4a). Active surveillance performance exhibited a linear relationship with population density, whereas passive surveillance provided high detection probabilities when population density was higher than 0.1 individuals/km^2^, but rapidly decreased its detection probability for lower density values (Figure 4b).

**Figure 5:**
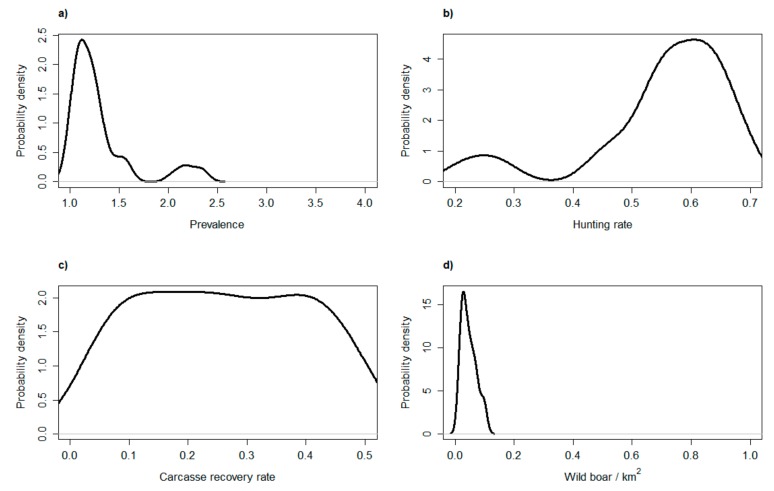
Frequency distribution of four parameter values (endemic prevalence (**a**), hunting rate (**b**), carcass recovery rate (**c**), population density (**d**)) corresponding to the simulated scenarios in which active surveillance was more effective than passive surveillance in revealing an African Swine Fever (ASF) outbreak.

Under the hypotheses of 50% carcass detection, active surveillance was the best detection method when the endemic disease prevalence was lower than 1.5%, when hunting rate was >60%, and when population density was lower than 0.1 individuals/km^2^ (Figure 5). In such a situation, though, the absolute probability to detect the disease is very low with both methods (Figure 4b), and finding almost every carcass is the only way to ensure virus detection. Additionally, the sensitivity analysis shows that carcass search effort is the sole parameter that increases proportionally the chance of ASF virus detection.

Sensitivity values of all parameters are shown in Table 4. In the case of passive surveillance, increased lethality and force of the infection (the rate at which susceptible individuals acquire the disease, λ [8]) reduced the time of detection in a less than a proportional manner. A 10% increase of the above-mentioned parameters reduced detection times of 2.4 and 2.2%, respectively. Intuitively, the increased proportion of carcasses found in the forest proportionally decreased the time necessary to detect the virus. An increase in the hunting effort mathematically decreased the probability to detect the virus through passive surveillance (−0.6%). The 10% increased hunting effort increased the efficiency of active surveillance in a non-proportional way, allowing a 3% reduction in the time necessary to reveal the disease. Also, the increased 10% in the force of the infection (λ) non-proportionally increased the performance of active surveillance, providing an 8.6% reduced time to detect it through both antigens and antibodies.

## 4. Discussion

Passive surveillance is the most effective way of detecting the presence of ASF in wild boar and to follow the epidemic phase in a wild boar infected population. Hence, surveillance aimed at early detection and to follow the first epidemic phase should not be based on sampling of hunted animals.

Among the parameters that drive surveillance activities, only hunting and carcass finding can be modified by management authorities. Any reasonable increasing of hunting, though, will not achieve the same probability to detect the virus than the one exhibited by passive surveillance. Our results showed than only by hunting more than 60% of the wild boar post-reproductive population, active surveillance would achieve the same probability to detect the virus than the one exhibited by passive surveillance. Such hunting effort is not considered achievable in short term and could be even counter effective, as it would likely increase animal movement due to disturbance [9]. When the infection was simulated in a small population, only passive surveillance had a 100% detection probability, whereas active surveillance exhibited a much lower detection capacity. In practice, at hunting ground level, if passive surveillance is not fully implemented, hunters might be unaware to hunt in an infected area, even when the infection is already/still present. In larger populations, the epidemiological patterns are easier to be monitored because of the increased chance that at least one ASF infected carcass will be found, or an infected individual will be shot. Still, the highest probability to detect the infection was associated to passive surveillance.

In both cases, simulations showed that disease detection during the endemic phase is unlikely and rather problematic, regardless of the method used. The pattern highlighted was consistent with the epidemiological findings in the field, where the virus seems to have locally disappeared, but it is again newly detected after several months [4]. Only in relatively large endemic areas a steady detection of the virus is likely to be observed in both dead and hunted animals, even if most of the virus detections are still from dead animals. At low wild boar density, the virus detection (both passive and active) is mainly driven by stochasticity. In such a scenario, only a progressive increase in carcass detection rate, inversely correlated with wild boar density, ensures the efficiency of surveillance. Variability of seasonal hunting, depopulation attempts, rewards for reporting dead wild boar, have the potential to modify the patterns highlighted by the model, thus also modifying the efficiency of both passive and active surveillance. It is worth to underline that the sensitivity analysis identifies the search of dead wild boar as the sole parameter having a linear connection between field effort and surveillance efficiency.

The use of serology, according to the model outcome, is of poor value (if any) in the first phases of the infection. Active surveillance aimed at detecting Abs positive animals, though, could be useful or even preferable at a later stage of the endemic phase; however, since Abs can be detected even in the absence of virus circulation, the use of serological surveillance has to be further fine-tuned considering all the epidemiological factors determining the possible surveillance outcomes.

Therefore, to enhance surveillance efficiency in each of the epidemiological phases characterizing ASF in wild boar, an effort should be made to promote reporting of dead wild boar, by maintaining or increasing awareness amongst the persons that in any way could report wild boar carcasses to the competent authorities; this is particularly important in those areas where ASF eradication is almost achieved and where surveillance has the main task to demonstrate the absence of the virus.

## 5. Conclusions

At very low wild boar density (i.e., 100/1000 km^2^), the virus is hardly detected through the usual surveillance strategies (both passive and active); in such epidemiological scenario, the available resources should be addressed at increasing passive surveillance with the aim of understanding where the virus is still present and removing infected carcasses. A low wild boar density, in fact, carcasses are the main responsible for maintaining the chain of infection in the wild.

## Figures and Tables

**Table 1:** Description, symbology and values of the main parameters used to build an epidemiological model of the African Swine Fever (ASF) in a simulated wild boar population.

Parameter	Symbol	Description	Rates
Lethality	γ	% of infected wild boar that die due to ASF	95% after five days from the infection = 0.19 day^−1^
Recovery rate	θ	% of infected individuals who survive and develop immunity	5% after 15 days from the infection = 0.0033 day^−1^
Hunting rate	ε	% of hunted wild boars in 365 days	40% of the post reproductive population in one year = 0.0011 day^−1^
Recruitment	μ	% of new-borns who enter the population	100% increase in a 30-days period = 0.033 day^−1^
Carcass recovery rate	ϕ	% of dead wild boars found and recovered	10% and 50%

**Table 2:** Relative performance of passive and active surveillance in the early detection of African Swine Fever (ASF) in wild boars, as resulting from a compartmental model. The results refer to the first year after the first disease outbreak.

Wild Boar Pop. Size	Early Detection (Days 1–100)						Epidemic Phase (Days 101–365)					
	Probability to Detect the Disease		Day of First Detection		Days between Detections		Probability to Detect the Disease		Day of First Detection		Days between Detections	
	Passive (ϕ ^a^ = 0.1)	Active	Passive (ϕ ^a^ = 0.1)	Active	Passive (ϕ ^a^ = 0.1)	Active	Passive (ϕ = 0.1)	Active	Passive (ϕ = 0.1)	Active	Passive (ϕ = 0.1)	Active
100	100%	43%	41	78	12	90	100%	62%	49	178	43	242
400	100%	87%	24	57	4	61	100%	100%	34	76	10	149
1000	100%	99%	15	35	1	24	100%	100%	31	54	3	58

^a^ Probability to detect a carcass of a wild boar dead for ASF.

**Table 3:** Average number of African Swine Fever (ASF) detections obtained through active and passive surveillance, as resulting from a compartmental model of the disease dynamics in a wild boar population. The results refer to the first year after the first disease outbreak.

	Early Detection (Days 1–100)				Epidemic Phase (Days 101–365)			
	Passive Surveillance		Active Surveillance		Passive Surveillance		Active Surveillance	
Wild Boar Population Size	N. Carcasses (ϕ = 0.1)	N. Carcasses (ϕ = 0.5)	N. Shot Wild Boar ASFV Positive	N. Shot Wild Boar Ab Positive	N. Carcasses (ϕ = 0.1)	N. Carcasses (ϕ = 0.5)	N. Shot Wild Boar ASFV Positive	N. Shot Wild Boar Ab Positive
100	6.9	27.5	0.7	0.01	4.4	16.2	0.4	0.08
400	28.5	112.1	2.8	0.07	17.2	64.2	1.7	0.3
1000	71.1	279.9	6.8	0.2	43.3	160.9	4.4	0.8

**Table 4:** Results of a sensitivity analysis performed on a compartmental model of African Swine Fever (ASF) in wild boars, following the method of Keeling and Gilligan [7]. Sensitivity values refer to the variation in the time necessary to first detect the disease, corresponding to a 10% variation in each parameter value.

Parameter	Passive	Active (ASFV Positive)	Active (Ab Positive)
Force of infection (λ)	0.22	0.86	0.86
Lethality (γ)	0.24	−0.06	−0.04
Hunting effort (ε)	−0.06	0.30	0.30
Recovery rate (θ)	0.00	0.00	0.40
% found carcasses (ϕ)	1.00	0.00	0.00
